# Dental Hints Department

**Published:** 1906-04

**Authors:** B. H. Teague

**Affiliations:** Aiken, S. C.


### DENTAL HINTS DEPARTMENT

Conducted by B. H. Teague, D. D. S., Aiken, S. C., to whom all contributions to this department should be sent.

If a "cold in the head" be taken in its inception, a 10-grain dose of iodid of potassium will promptly dispel it.—*Med. World.*

In biliousness, if stools are dark, podophyllum is indicated; if the stools are light in color, calomel is the drug indicated.—*Med. World.*

In preparing cavities for amalgam the best results were to be obtained by making flat seats for it to rest upon and parallel walls extending from these seats.—*Dr. C. N. Johnson, Review.*

Newspaper publishers complain that ethical dentists do not contribute to their welfare by advertising. It is not unethical to keep a card, with simply name and address, running in a newspaper or magazine.

Formalin, either in paste or fluid should not be used in dressing teeth after the nerves have been recently devitalized. Severe pain and soreness is often caused by its use. It is indicated only in putrescent roots.

"The Way of the Transgressor Is Hard."—With dental students operating on their teeth to pass a practical examination, the convicts in the Idaho penitentiary will renew their efforts to secure a pardon.—*Portland Telegram.*

Porcelain Inlays; the Matrix.—The metal should never be annealed and the matrix introduced into the cavity after the final burnishing, as it is too easy a matter to distort the matrix in so doing.—*Craig M. Work, Dental Summary.*

In repairing with vulcanizable gutta percha, or waxable rubber, it is well to pack into this waxable material, after it has been forced into the holes and undercuts, a greater bulk of regular unwaxable rubber. Greater strength to the patch may be thus secured.

Shrinkage in Rubber.—The amount of shrinkage of rubber in vulcanizing depends upon its composition. Pure rubber and sulphur shrinks the most; red rubber less; pink rubber still less, in proportion to the amount of coloring matter.—*Geo. B. Snow.*

Preparing Sensitive Cavities.—The first application of the burr can be made absolutely painless in the most highly sensitive cavity by simply taking ethyl chlorid on the burr point and bringing it quickly into contact with the tooth.—*J. M. Gale, British Dental Journal.*

Artificial Teeth Caused Death.—Artificial teeth caused the death at Kokomo, Ind., of Mrs. Sarah Coston, aged 58. A defective plate resulted in the abrasion of the skin and septic poison. All the bones on one side of the head became affected. Her suffering was intense.—*Summary.*

Lubricants.—An important fact to be borne in mind is that oil in a closely fitting long-bearing is a lubricant only when present in small quantity; in large quantity it acts as an adhesive. Little oil lets the machine go; much oil holds it fast.—*Dental Office and Laboratory.*

Richmond Metal.—Do you use this valuable adjunct to many operations? If not, try it. Its advantages over other impression metals is its low fusing-point—160 degrees—52 below boiling water, enabling you to run a model over a gutta-percha impression.—*J. P. Root, Kansas Den. Jour.*

It is doubtful whether teething alone can cause convulsions, but in the presence of rickets, and alimentary disturbance, the additional irritation of teething may cause additional alimentary disturbance, and a convulsion result from reflex alimentary trouble or alimentary toxæmia.—*British Med. Jour.*

Vulcanizer Exploded.—By the explosion of a vulcanizer in his dental office December 26, Dr. Fred Hale, of Hudson, Mich., sustained severe injuries about the head and face and it is feared the sight of his right eye is destroyed. The injured man was taken to the Ann Arbor Hospital.—*Saginaw Herald.*

Early Regulation.—No matter what the operation, early treatment is always advisable, the less number of permanent teeth erupted making the operation easier and shorter because of the lesser resistance to be overcome, and because of the cartilaginous nature of the process at early age.—*Herbert A. Pullen, Items of Interest.*

Multiple Warts.—Dr. Mantelin recommends the following prescription in the treatment of multiple warts:

℞ Chloralis hydratis, acidi acetici ............āā ʒiss

Acidi salicylici, spts. etheris ...............āā ʒi

Collodii ....................................ʒiv

Apply locally to the warts twice daily.—*Med. Chir. Jour.*

How to Keep Well.—Nature is always and forever trying hard to keep people well; and most so-called disease—which word means merely lack of ease—is self-limiting, and tends to cure itself. If you have no appetite, do not eat. If you have appetite, do not eat too much. Be moderate in the use of everything, except fresh air and sunshine.—*Good Health.*

Expansion of Rubber.—Black rubber expands the most by heat; pink the least. Pure rubber is the most apt to become spongy if thick pieces are over-heated in vulcanizing. If stale from long keeping blow-holes or bubbles will be developed under the surface, even at low heat. Always buy fresh stock.—*Geo. B. Sword, Brief.*

A satisfactory cement for mending broken china, and the knowledge of which will come in play about the doctor's office, is as follows: Into a saturated solution of gum arabic in water, stir sufficient plaster-of-paris to make a thick paste. Apply with a soft brush to the cleaned edges of the fracture and press firmly together for a few minutes. It holds well.—*Med. World.*

"Strengthened Dental Rubber."—A toughened rubber, free from all brittleness and of very great strength is produced by a process of rolling a sheet of metal gauze between two sheets of rubber, the metal being of a quality not acted upon by the constituents of the rubber. Used in one piece, roughly cut to the shape of the plate.—*Dental Record.*

Sealing the Dental Tubuli.—The right quantity of cement, mixed to a proper consistency, placed beneath gold foil in the cavity, and subjected to a pressure sufficient to weld the gold, will so perfectly seal the dental tubuli that we may entirely prevent the black rim of discoloration so prevalent between cohesive gold and the tooth substance, when the cavity is unlined.—*F. L. Trickey, Dental Review.*

For Cavities in Porcelain Inlays.—For low fusing body, I use gold pellets rolled and shaped as required. For high fusing porcelain, graphite, or lead pencil plumbago, is well adapted, both, under the heat used, holding their shape and after fusing being easily removed. The cavity side of the finished inlay is, of course, etched in addition, as usual.—*Dr. C. B. Rohland, Brief.*

I was told once how to carry a very small portion of cement. Take a very delicate broach and either make a little ring at the end of the broach, or bend the point at an angle of 45 degrees, or 90 degrees—just a little bend that you would almost have to take a magnifier to see—and you will find you can carry a little globule on the end of the broach. Otherwise it would tend to run back.—*Dr. W. V. B. Ames.*

Plaster of Paris and Paraffin Wax an Excellent Carving Material.—If you will take ordinary paraffin wax, melt it in a large spoon, and stir plaster of Paris into it while it is in a molten condition, you will find that it will make one of the nicest carving materials that you have ever used. Make it plastic by heating, apply it to the primary crown, and allow the patient to bite into it, and you have the cast.—*J. Q. Byram, Dental Register.*

A Source of Failure in Approximal Fillings.—The removal of unsupported enamel is an absolute necessity, whether upon the occlusal surface, upon the buccal or lingual walls, or upon the gingival margin, not only because it has not sufficient strength to withstand the stress of mastication, but because it does not possess sufficient strength to build and condense gold against without fracturing or breaking under the force of condensation.—*I. F. Wallace, Dental Era.*

Porcelain Inlays: Adhesion.—It has been demonstrated time and again that cement will not adhere to a glazed surface as well as to an etched surface. I have found from experience that in all cases where an inlay has dropped out of a cavity you will find that the cement adhered to the surface of the tooth, and not to the inlay itself. I do not attempt to groove my inlays any more on account of the danger of serious damage, and I find it is not necessary.—*J. E. Nyman, Dental Summary.*

Flux for Soft Soldering.—Pieces of zinc are dissolved in hydrochloric acid until the acid is saturated. The resultant solution of zinc chloride is mixed with an equal amount of a mixture consisting of aqua ammonia and alcohol. After standing a few days the solution is filtered and ready for use. The so-called Miller's soldering fluid consists of a solution of phosphoric acid in eight parts of water, to which one part each of lactic acid and glycerine has been added.—*The Dental Era.*

Silver Nitrate.—Bryan of Basel has inaugurated a system of dental prophylaxis, in which he strongly advocates the application of a 40 per cent. nitrate of silver solution upon tooth surfaces slightly affected by the earlier stages of caries. With intervals of three months this process is to be repeated. He is very enthusiastic about his results, believing that the stimulating effect of the application causes the pulp to retract by depositing secondary dentin in the crown chamber.—*Hermann, Prinz, Dental Digest.*

Dental Salesmen Organize.—The dental salesmen of the United States have organized the National Association of Dental Salesmen. The officers are: President, C. A. C. Kelly, Buffalo; Vice-President, Charles J. Hood, Pittsburg; Secretary, Edwin R. Davis, Buffalo; Treasurer, C. Guy Calder, Pittsburg; Directors, L. M. Beardsley, of St. Louis, Oscar Bieg, of Philadelphia, Frank Mills, of New York City, H. G. Stephens, of Kansas City, N. M. Stackhouse, of Buffalo.—*Buffalo Commercial.*

Extraordinary Dentistry.—Perhaps the greatest dental operation on record was performed upon an elephant in the City of Mexico. The aching tooth was 12 inches long and four inches in diameter at the root. After Mr. Elephant had been securely fastened with chains his mouth was pried open and a quantity of cocaine applied to deaden the pain. When this was done, a hole was bored through the tooth and an iron bar inserted. Then a rope was twisted around the bar and four horses attached.—*La Crosse Press.*

The Porcelain Crown.—I would advise the use of the 25 per cent platinum solder when attaching the floor of the band and the dowel to the floor, especially if using a high fusing body. If the dowel can be raised up a little above the floor it is stronger as regards the possibility of the crown on the dowel. If the end of the dowel is flattened out on an anvil, spreading it to a dovetail, and given a little bend backward so as to get it back with the lingual cusp, it will greatly strengthen the crown.—*F. H. Berry, Dental Review.*

One of the greatest evils in dental practice is that of long credits, and yet with traditions and customs as they are today it is frequently necessary to extend credit to most patients. As it is awkward and embarrassing to ask for a settlement after each sitting, the extension of credit cannot be avoided. However long credits are exasperating in practice, and a dentist should so conduct his practice that it will be apparent to his patients that he is opposed to unlimited credit.—*Dr. G. F. Burke, Register.*

Chronic Alveolar Abscess.—Persons suffering from chronic alveolar abscesses are always more or less liable to some constitutional disturbances in which it would be difficult to trace the initial cause to this almost unrecognizable condition. From the fistulous opening broken-down tissue cells are discharged into the oral cavity and become constituents of the fluids of the mouth, contaminating the secretions in such a way as to prevent its physiological action on the food that is introduced in the stomach and intestines to compensate for the waste that goes on in the body.—*Geo. W. Cook, Dental Digest.*

Sterilising Root Canals.—In from ten to fifteen minutes the electrolytic action of a constant galvanic current of from one to two milliamperes suffices for sterilising completely root canals inaccessible to instrumentation. Injecting a 75 per cent aqueous solution of common salt, thoroughly saturating the canal contents, gives a good conductor. If the anode or positive pole is made of platinum it will deposit chlorine, oxygen and hydrochloric acid, these ions promising a decided anti-bacterial effect.—*Dr. Kurt Hoffendahl, "Dental Cosmos."*

Inlays: the Gold Matrix.—It has been suggested that the matrix be formed of No. 36 gold plate, and that the portion which comes in contact with the margins, and for a considerable distance below, be left upon the inlay. After the cement is thoroughly hardened the surplus gold that overhangs the margins is to be cut off and the remaining gold burnished to cover up the line of cement. The advantages are that the heating is so heavy that it will not pull in fusing the porcelain, and it makes a more perfect union between the inlay and the tooth. I have found this method of great value.—*F. T. Van Woert, Dental Cosmos.*

THE PORCELAIN CROWN.—I would advise the use of the 25 per cent platinum solder when attaching the floor to the band and the dowel to the floor especially if using a high-fusing body. If the dowel can be raised up a little above the floor it is stronger as regards the possibility of the crown tipping on the dowel. If the end of the dowel is flattened out on an anvil, spreading it to a dovetail, and given a little bend backwards so as to get it back with the lingual cusp, it will greatly strengthen the crown.—*F. H. Berry, Dental Review.*

HYGIENIC FILLINGS.—The sealing of the tubuli is the one important feature in the preservation of tooth structure. We now have a material for this purpose that is better than cement; material that is ready for instant use, that requires no mixing, that sets instantly, that is non-conducting, adhesive, and antiseptic, thus forming the ideal lining; it firmly anchors the first piece of gold, upon which the rest of the filling may be packed without danger of loosening or dislodgment. This material is Gold Anchor Intermediary, composed of thymol, dammar, baric sulphate and oil of cloves.—*Alison R. Lawshe, Items of Interest.*

THE ANATOMICAL ARTICULATOR IN CROWN AND BRIDGE WORK.—I wish to lay particular stress upon the use of the anatomical articulator in crown and bridgework, and I wish to go one step farther. In bridge work I advocate taking impressions of the entire mouth and articulating models of the entire mouth. You will find that it is seldom possible to take impressions of half the mouths, articulate them on an anatomical ariculator, and obtain the same accurate results that you can obtain if you have casts of the entire mouth, and I wish to emphasize that point, because it is neglected. I believe if we would use the anatomical articulator more in our crown and bridge work we would have fewer bridges loosening in a few years.—*J. Q. Byram, Dental Register.*

A WATER-TIGHT GOLD FILLING.—Prepare cavity as for an inlay and then give a return from either by grooves or dovetails. Dry the cavity and line with thin cement. Cut a piece of No. 30 or No. 40 gold foil as though for taking an impression for an inlay and put it down in the cavity in the usual impression method. This gives a cavity lined with gold, with cement in every possible irregularity. Fill with any cohesive gold which the operator is in the habit of using, beginning first with the undercuts where the lining may be spilt, condensing thoroughly with hand plugggers and finishing in the usual way. The method is applicable to the tiniest gold filling or the largest contour, and makes gold filling easy and sure.—*W. Thompson Madin, British Dental Journal.*

When patients of different social position in life present themselves to us, it is then for us not to feel a preference for the intelligent and well-moneyed person; but when we consider that one of limited means has placed himself in our hands with the full knowledge that our fees are greater than others might have asked of him, when they have expressed their confidence in our ability, and our services are worth more, it is certainly up to us to give them our best efforts. It is like robbing them of their life's blood to do otherwise. However, we should make a difference. That might be all right in communities, in large cities, where the wealth is reckoned by thousands or millions, but in smaller cities or country towns to attempt to do it in that line is certainly to invite trouble.—*Dr. Dunn Warren, Summary.*

Girl's Bite Is Often Deadly.—Prof. W. D. Miller, of the University of Berlin, sent shivers down the backs of the students at Wesleyan, Middletown, Conn., when he announced in a lecture, delivered October 11, that the bite of a girl would often bring a quicker and more horrible death than the bite of a serpent. Professor Miller, who has made a special study of the bacteria of the mouth, said that only a short time ago he experimented on a girl in Germany and found that an arrow dipped in saliva from her mouth would send its victim in death throes more terrible than one dipped in the venom of the most deadly snake. Professor Miller said that there was a lesson in this for dentists.—*Exchange.*

Cavity-Preparation for Inlays.—Complaint is frequently made that porcelain inlays are liable to come out of labial cavities. The chief trouble is that the cavity-preparation is imperfect. A cavity with a rounded base will not do. Make the axial wall flat and make an angle between this wall and the surrounding walls. It need not be a sharp right angle, but it may be very nearly this, and still permit of the removal of the matrix. The tendency to make rounded, saucer-shaped cavities is accountable for most of these failures.—*Frank E. Cheeseman, Review.*

A Method of Manipulating a Positive Matrix.—In describing what I have termed a method of manipulating a positive matrix for inlay work, I always prepare the cavity in such a manner that an impression can be taken that will readily draw. From the impression taken in modeling composition the matrix is first outlined over it in order that it may be readily introduced into the cavity without puncturing or warping, and with sufficient margin all around the border. With cotton pellets and burnishers I thoroughly form the matrix to cavity, removing all cotton I force the modeling compound impression, first taking the precaution to slightly oil it, into the cavity and over matrix and firmly swage by use of fingers or suitable appliances. Removing impression and matrix together I invest in a small platinum tray in three parts powdered silex to one of plaster mixed with water to a proper consistency. The investment is practically non-shrinkable, and will withstand the heat for any number of bakings required to complete the inlay. This method will give you a matrix that is in positive contact with all points of the cavity; eliminate direct handling of matrix with fingers or instruments up to the completed inlay.—*F. S. Morrison, Dental Summary.*

Have you ever replaced broken facing by Bryant's method? Not the gold nuts, but by connecting the two pins with tinner's solder, filing them into a square shape, cutting a corresponding square box shape hole in gold, backing and cementing into position. An improvement over his way is to grind facing to fit; make an oblong box, open at each end, out of steel matrix metal; place over pins, set tooth on stove, pin side up, with box in position, and a piece of Richmond metal on top. A few moments of slight heat and your metal flows into box, around pins, and you have your tooth ready to file to fit the slot in backing; cement into place, and, with warm burnisher melt the metal on lingual surface, smoothing down into all inequalities, and the result is what might be termed a riveted retention. Try it.—*Dr. J. P. Root, Kansas Den. Jour.*

A Water-Tight Gold Filling.—Prepare cavity as if for an inlay and then give a retention form either by grooves or dovetails. Dry the cavity and line with thin cement. Cut a piece of No. 30 or No. 40 gold foil as though for taking an impression for an inlay and put it down in the cavity in the usual impression method. This gives a cavity lined with gold, with cement in every possible irregularity. Fill with any cohesive gold which the operator is in the habit of using, beginning first with the undercuts where the lining may be split, condensing thoroughly with hand pluggers and finishing in the usual way. The method is applicable to the tiniest gold filling or the largest contour, and makes gold filling easy and sure.—*W. Thompson Madin, British Dental Journal.*

FLASKING SO AS TO PREVENT RED RUBBER GETTING THROUGH THE PINK.—Pink rubber is much weaker than the red and in investing the model and teeth in the flask, I use a shallow one (the anchor flask) and only allow the soft plaster to come *up to the base plate and not over it,* then finish the investment in the usual manner. In separating you will find you can pack all the rubber in one side, pink first, cut in v-shaped pieces, then this pink can be backed with red rubber and greatly strengthened without the danger of the red running through, your plate being less liable to be broken. This can be done with ease after one or two trials. The teeth are almost entirely in red rubber.—*Dr. R. H. McLaughlin, Charlotte, N. C.*

Quite frequently in blind abscess cases, after the pus formation has been checked, we have a weeping of serum from the canals. I find this condition more often associated with the superior laterals than with any other teeth, although it is sometimes found in connection with all teeth from which abscesses have been treated. An excellent remedy to use here is eucalyptol, to which thymol has been added in the following proportion:

| | | |
|---|---|---|
| ℞ | Eucalyptol | f ʒ j |
| | Thymol | gr. X |

M. Sig: Use as directed.

If this remedy fails to check the secretion and the liquid is *serum* (not pus), no hesitancy need be had in filling the root although the canals should be moist.—*Dr. J. P. Buckley, Review.*

Porcelain inlays are beautiful, artistic and harmonious in color, and should quite properly usurp the place of gold fillings in exposed tooth surfaces whether they prove as durable or not, but they have been tried too short a time to enable us to decide that they are better than, or as good as, gold in places where its color does not offend. Where no strain of mastication is brought to bear upon them the only limit to the permanence of porcelain inlays lies in the durability of the cement which holds them in position. That there is a gradual dissolution or wasting away of this cementing medium is perfectly certain, for we see it constantly under other conditions of its employment in the mouth. It is the same material in each case and subject to the same influence, so that those who claim that the disintegration of the cement does not take place when placed between porcelain and tooth-substance are simply deceiving themselves.—*Ex-Ed. Stomàtològist.*

Care of the Gates-Glidden Drill When Out of Use.—No less important than the care of the drills while in use in the care of them out of use. When they are frequently in use their sharpening is an item that should never be neglected, as a blunt drill, besides being ineffectual, is a source of annoyance to both operator and patient, and is the cause of many mishaps excused on the plea of "faulty drills." The method of sharpening them which I will describe, although familiar to many, may still be new to some.

With the use of a piece of oilstone shaped like a pocket-knife blade (say three inches in length, and with a sharp edge), it is a comparatively easy matter to bring in turn each blade of the drill to an edge, examining the drill frequently during the process under a magnifying glass. It is essential to good work that the stone edge be kept sharp. To accomplish this, obtain a piece of hardwood say three inches long and two inches in width, making sure that it is straight and perfectly level. Now take a strip of sheet lead two inches wider than the wood, but the same length or longer, so that it may be continued over each end; tack it over the sides and ends (if necessary) to the wood, after having adjusted it evenly. Upon the surface thus formed sprinkle some No. 1 emery, and rub the stone lengthwise, without oiling or wetting the surface. To keep the stone in perfect condition this process should be continued after every dozen drills. As to the time required for sharpening the stone, three minutes usually suffices.

When utterly worn out the drills can still be made to render useful service by grinding them to a spear-point upon the lathe stone. In this shape they are very useful in alveolar abscess, where it is to be approached from the outside, through the alveolar process.—*Dr. S. J. Fernandez, Extract, Cosmos.*

